# Treatment strategies and LDL cholesterol target attainment in patients with statin intolerance: Insights from the multicentre statin intolerance registry

**DOI:** 10.1016/j.ajpc.2025.100953

**Published:** 2025-03-01

**Authors:** Paulina E. Stürzebecher, Julius L. Katzmann, Ionna Gouni-Berthold, Christina Mateev, Ole Frenzel, Ulrike Schatz, Andrea Baessler, Wolfgang Koenig, Stephan H. Schirmer, Irina Müller-Kozarez, Oliver Weingärtner, Ursula Kassner, Ulrich Laufs, Statin Intolerance Registry investigators

**Affiliations:** a Klinik und Poliklinik für Kardiologie, Universitätsklinikum Leipzig, Leipzig, Germany; b Center for Endocrinology, Diabetes and Preventive Medicine, University of Cologne, Faculty of Medicine and University Hospital Cologne, Cologne, Germany; c Department of Internal Medicine III, University Hospital Carl Gustav Carus, Technische Universität Dresden, Dresden, Germany; d Department of Internal Medicine II, University Hospital Regensburg, Germany; e German Heart Centre Munich, Technical University Munich, German Centre for Cardiovascular Research (DZHK), Partner Site Munich Heart Alliance, Munich, Germany; f Institute of Epidemiology and Medical Biometry, University of Ulm, Ulm, Germany; g Kardiopraxis Schirmer, Kaiserslautern, Germany; h University Hospital Jena, Department of Internal Medicine I, Jena, Germany; i Charité – University Medicine Berlin, Clinic for Endocrinology and Metabolic Medicine, Berlin, Germany

**Keywords:** Muscle symptoms, Cardiovascular risk, Ezetimibe, Bempedoic acid, PCSK9 inhibitors, Statin

## Abstract

**Objective and methods:**

Statin intolerance (SI) is an important cause of insufficient low-density lipoprotein cholesterol (LDL-C) target attainment. Contemporary treatment strategies and symptoms in patients with SI are incompletely understood. We report baseline lipid-lowering therapies (LLTs) and LDL-C target attainment in the Statin Intolerance Registry, an observational, prospective, multicenter study that recruited 1,111 patients with SI between 2021 and 2023 in Germany.

**Results:**

The mean age was 66.1 (SD 9.9) years, 57.7 % were female. At study inclusion, 83.1 % received at least one LLT, and 47.0 % received combination LLT. A higher number of LLTs was associated with lower LDL-C, lower systolic blood pressure, more atherosclerotic disease, more elevations of creatine kinase and liver enzymes but not with impaired quality of life as measured by EuroQol (EQ-5D-5L). PCSK9 inhibitors were most frequently prescribed (48.0 %), followed by ezetimibe (39.2 %), statins (26.9 %), most commonly rosuvastatin, and bempedoic acid (25.4 %). Patients who had been prescribed multiple statins before were more likely to take a statin at baseline. Patients on a statin, even at low intensity, had lower LDL-C levels compared to patients without statin therapy (mean [SD] 2.4 [1.2] vs. 2.9 [1.6] mmol/L, *p* < 0.001). Significantly more men than women achieved the LDL-C target (21.7 % vs. 11.4 %, *p* < 0.001, total cohort: 15.8 %).

**Conclusion:**

LDL-C target attainment is low in patients with SI, especially among women, despite high cardiovascular risk. The use of a greater number of LLTs, including statins, is not associated with reduced quality of life but is associated with lower LDL-C levels.

## 1 Introduction

The inability to tolerate doses of statins that lead to sufficient reductions of low-density lipoprotein cholesterol (LDL-C) concentration, or statins at all, denoted “statin intolerance” (SI), is one important reason for suboptimal LDL-C target achievement as recommended by current guidelines [1,2]. SI affects one tenth of patients taking a statin and is associated with increased risk of atherosclerotic cardiovascular disease (ASCVD) events [3,4]. The management of patients with SI remains challenging [[5], [6], [7], [8], [9]]. Data from prospective registries on contemporary treatment strategies and LDL-C target attainment in patients with SI are lacking.

## 2 Methods

The prospective, observational, multicentre Statin Intolerance Registry (SIR) enrolled patients from May 2021 to June 2023 at 19 participating sites in Germany. SI was defined as intolerance of at least two statins (1) at any dose or (2) the inability to increase the weekly statin dose beyond 70 mg of atorvastatin, 140 mg of simvastatin, pravastatin, or lovastatin, 35 mg of rosuvastatin, or 280 mg of fluvastatin. The design of the SIR has been described in detail previously [10]. This analysis reports treatment strategies and LDL cholesterol target attainment at inclusion. Each local Ethics Committee approved the study, written informed consent was obtained from all participants. The study is registered at ClinicalTrials.gov NCT04975594. This non-interventional study collected data based on standard of care. Treatment decisions were made by the attending physician and were not influenced by study participation. Baseline data collection encompassed demographic parameters, comorbidities, current treatment regimens, previous statin therapies and any associated adverse events, and laboratory parameters. The term “PCSK9 inhibitors” encompasses the monoclonal antibodies evolocumab and alirocumab and the small-interfering RNA inclisiran.

“Low-intensity statin” was defined according to the CLEAR Outcomes study. „Low-intensity statin“ denotes daily doses of statins below those specified in the inclusion criteria [11]. High-dose statin was defined as atorvastatin 40–80 mg and rosuvastatin 20–40 mg daily [12].

In addition, standardized questionnaires regarding depression (PHQ-9) [13] and health-related quality of life measured by EuroQol (EQ-5D-5 L) [14] were completed by the patients. “Low quality of life” was defined as EQ VAS scale values below the median score of 70. Muscle pain during statin therapy was assessed using a numeric rating scale (NRS), ranging from 0 to 10, where 0 was defined as "no pain" and 10 as "unbearable pain" [16]. “High pain intensity of SAMS” was defined as > 7 points on the NRS. Patients reporting muscle pain while on statin therapy were further evaluated using the statin-associated muscle symptom clinical score (SAMS-CI) [17]. A SAMS-CI score of lower than 7, 7 or 8, or higher than 8 suggests that the muscle symptoms are unlikely, possibly, or probably associated with statin therapy, respectively. Cardiovascular risk was determined according to the 2019 ESC/EAS dyslipidemia guidelines [2]. Study data were collected and managed using REDCap electronic data capture tools hosted at the Center for Clinical Studies (ZKS), Leipzig, Germany [15]. Group differences in continuous variables were assessed with Student's t-test, and binary variables with the Chi-squared test. Univariate logistic regression and one-way anova was applied to analyse the association of an increasing number of lipid-lowering drugs and variables selected based on clinical plausibility. Bonferroni correction was applied for multiple testing. Statistical analyses were performed using Stata/MP 18.0.

## 3 Results

A total of 1111 patients were included. The mean age was 66.1 (9.9) years, 57.7 % were female. ASCVD was present in 88.0 %, 27.4 % had a diagnosis of familial hypercholesterolaemia (FH) (Table 1). At study inclusion, 16.9 % did not receive any lipid-lowering therapy (LLT), and 36.1 %, 34.3 %, and 12.7 % received one, two, or at least three lipid-lowering agents, respectively. PCSK9 inhibitors (PCSK9i) were most frequently prescribed (48.0 %, inclisiran representing 9.2 % of all PCSK9i), followed by ezetimibe (39.2 %), statins (26.9 %), and bempedoic acid (25.4 %). Among statin prescriptions, 65.7 % were at low intensity, and rosuvastatin was most common (53.8 %), followed by atorvastatin (16.7 %). Patients on statin had lower LDL-C concentrations than those without (2.4 [1.2] vs. 2.9 [1.6] mmol/L, *p* < 0.001) and more often achieved the LDL-C target (18.4 % vs. 14.8 %, *p* = 0.14). Prior to inclusion, 27.6 %, 40.3 %, and 32.0 % had taken two, three, or at least four statins, respectively. An increasing number of statins taken was associated with a higher proportion of patients at LDL-C target (target achievement for two, three, and at least four statins taken 12.7 %, 16.3 %, and 17.7 %, respectively).

**Table 1:** Baseline characteristics stratified by number of lipid-lowering drugs, statin therapy and sex.

	Total (*n* = 1111)	No LLT (*n* = 188)	1 drug (*n* = 401)	2 drugs (*n* = 381)	3 or more drugs (*n* = 140)	Statin therapy (*n* = 299)	No Statin therapy (*n* = 812)	Women (*n* = 629)	Men (*n* = 468)
**Demographic data**									
Age (years)	66.1 (9.9)	65.4 (10.7)	67.2 (9.6)	65.9 (9.6)	64.2 (9.8)*	65.6 (10.4)	66.3 (9.8)	67.2 (9.2)	64.5 (10.7)†
Female	57.7	63.8	56.1	55.9	58.6	58.4	57.4	100	0
Systolic blood pressure (mmHg)	140.1 (18.4)	145.5 (16.8)	140.0 (19.0)	138.3 (18.2)	138.6 (18.4)†	139.5 (17.5)	140.4 (18.8)	139 (18.6)	138 (18.2)
Diastolic blood pressure (mmHg)	81.9 (10.4)	83.6 (10.8)	81.7 (10.5)	81.3 (9.8)	82.5 (10.8)	81.3 (10.6)	82.2 (10.6)	80.0 (10.6)	81.0 (10.1)
Body mass index (kg/qm)	27.4 (4.6)	27.6 (4.3)	27.4 (4.5)	27.1 (4.9)	27.5 (4.7)	26.9 (4.6)	27.6 (4.7)*	26.3 (4.9)	27.7 (4.2)†
**Laboratory parameters**									
Total cholesterol									
mmol/L	4.8 (1.7)	6.9 (1.4)	4.8 (1.5)	4.2 (1.2)	3.9 (1.2)†	4.4 (1.3)	5.0 (1.8)†	5.2 (1.7)	4.3 (1.5)†
mg/dL	187.2 (66.3)	269.1 (54.6)	187.2 (58.5)	163.8 (46.8)	152.1 (46.8)	171.6 (50.7)	195 (70.2)	202.8 (66.3)	167.7 (58.5)
LDL-C									
mmol/L	2.8 (1.5)	4.8 (1.3)	2.7 (1.3)	2.2 (1.0)	2.0 (1.1) †	2.4 (1.2)	2.9 (1.6)†	3.1 (1.6)	2.4 (1.4)†
mg/dL	109.2 (58.5)	187.2 (50.7)	105.3 (50.7)	85.8 (39.0)	78.0 (42.9)	93.6 (46.8)	113.1 (62.4)	120.9 (62.4)	93.6 (54.6)
HDL-C									
mmol/L	1.5 (0.5)	1.4 (0.4)	1.5 (0.4)	1.5 (0.5)	1.5 (0.4)	1.5 (0.5)	1.4 (0.5)†	1.6 (0.4)	1.3 (0.4)†
mg/dL	58.5 (19.5)	54.6 (15.6)	58.5 (15.6)	58.5 (19.5)	58.5 (15.6)	58.5 (19.5)	54.6 (19.5)	62.4 (15.6)	50.7 (15.6)
Triglycerides									
mmol/L	1.8 (1.1)	2.2 (1.4)	1.8 (1.0)	1.7 (1.0)	1.6 (0.9)†	1.5 (0.8)	1.9 (1.2)†	1.8 (1.0)	1.9 (1.2)
mg/dL	70.2 (42.9)	85.5 (54.6)	70.2 (39.0)	66.3 (39.0)	62.4 (35.1)	58.5 (31.2)	74.1 (46.8)	70.2 (39.0)	74.1 (46.8)
LDL-C at target (%)	15.8	0.0	30.3	44.6	25.1	18.4	14.8	11.4	21.7
**Cardiovascular risk factors**									
Hypertension	75.2	70.4	79.2	74.7	71.9	71.9	76.5	75.2	75.3
Diabetes	19.1	15.7	24.2	15.8	18.4*	14.4	20.9*	19.3	18.8
Active smoking	10.0	12.2	9.5	9.5	9.9	11.7	9.4	10.2	9.8
Family history of cardiovascular event	70.8	62.3	71.3	73.0	74.5*	72.6	70.1	73.8	66.6*
Atherosclerotic cardiovascular disease	88.0	75.9	90.5	88.7	95.7†	90.6	87.2	85.3	91.9†
Premature cardiovascular event or revascularization	27.4	11.2	25.7	31.2	34.0†	26.8	27.6	21.7	35.1†
Peripheral artery disease	7.7	6.9	8.2	7.1	9.2	7.4	7.9	6.7	9.2
Myocardial infarction	31.2	22.3	31.4	33.1	37.6*	29.8	31.8	23.4	41.9*
Stroke	9.9	8.5	11.0	9.5	9.9	7.7	10.7	10.5	9.2
Arterial revascularization procedures	50.2	34.6	52.9	52.3	58.2*	46.5	51.6	40.9	63.0*
FH	27.4	28.7	21.3	30.2	37.6	30.4	26.6	32.0	21.6
Suspected FH	17.3	6.9	20.0	20.2	15.6	21.1	15.9	18.0	16.4
**Comorbidities**									
Hypothyroidism	23.3	26.6	22.0	23.4	22.7	24.8	22.9	33.4	9.6
Orthopedic disease	53.0	50.6	61.5	48.8	42.9†	48.9	54.5	54.6	50.8
Cancer	10.9	11.8	10.3	14.3	13.2	10.6	12.9	12.3	12.3
Depression	10.3	7.5	10.5	10.2	13.5	10.4	10.2	9.8	10.9
Heart failure	7.6	1.8	11.1	9.6	7.4†	9.4	8.2	7.9	9.5
Rheumatic disease	9.9	10.6	11.9	9.0	5.7	5.8	11.4*	10.0	9.8
Chronic obstructive lung disease	4.1	5.3	4.2	3.7	3.6	5.0	3.8	3.1	5.5*
Chronic kidney disease	13.4	8.8	14.7	13.8	14.9	11.7	14.0	13.3	13.6
Psycho-social									
EQ VAS score	64.8 (12.1)	64.4 (18.5)	63.5 (18.1)	66.5 (17.1)	64.8 (17.8)	66.6 (18.0)	64.3 (18.2)	63.1 (18.2)	67.2 (17.7)†
PHQ-9 score	5.8 (4.4)	5.8 (4.2)	6.0 (4.3)	5.4 (4.6)	6.2 (4.7)	5.5 (4.3)	5.9 (4.5)	6.2 (4.5)	5.3 (4.2)†
**Other parameters of interest**									
Creatin kinase elevation while on statin	15.6	6.9	14.0	18.6	23.4†	19.4	14.2*	9.7	23.6†
Elevated liver enzymes while on statin	7.7	4.3	6.2	8.9	12.8*	10.0	6.8	8.1	7.0
Intensity of muscle pain while on statin	7.0 (1.8)	7.1 (1.8)	7.2 (1.6)	6.9 (1.9)	6.6 (1.7)*	6.5 (1.8)†	7.2 (1.7)†	7.1 (1.8)	6.8 (1.7)
SAMS-CI score	9.0 (1.8)	9.2 (1.7)	9.0 (1.7)	8.8 (1.9)	8.9 (1.8) †	8.7 (1.9)	9.0 (1.7)*	9.0 (1.7)	8.9 (1.9)

CI, clinical index; FH, familial hypercholesterolemia, HDL, high-density lipoprotein; LDL, low-density lipoprotein; LLT, lipid-lowering therapy; SAMS, statin-associated muscle symptoms.

Continuous values as mean (SD) and categorial values as %.

⁎ *p* < 0.05; † significant according to Bonferroni correction: *p* < 0.004 for continuous variables, *p* < 0.002 for dichotomous variables.

The most frequently prescribed monotherapy were PCSK9i with 57.4 %. Bempedoic acid was more commonly prescribed to women than to men (27.7 % vs. 22.1 %, *p* = 0.036), and PCSK9i more often to men than to women (53.2 % vs. 44.2 %, *p* = 0.003; Fig. Panel A). Medication-associated adverse events on non-statin LLT were reported by 57.4 % of patients (under ezetimibe, bempedoic acid, PCSK9i and medications other than LLT in 44.2 %, 23.7 %, 13.3 %, and 24.6 %, respectively). SIR participants reported an average of 6.8 (SD 7.6) physician visits in the 12 months prior to study inclusion related to hyperlipidaemia.

Women were more likely not to receive any LLT (18.3 % compared to 14.3 % of men), had significantly higher LDL-C concentrations compared to men (3.1 [1.6] vs. 2.4 [1.4] mmol/L *p* < 0.001; Fig. Panel B), and achieved the LDL-C target to a lower extent (11.4 % vs. 21.7 %, *p* < 0.001; Fig. Panel C).

An increasing number of lipid-lowering agents was associated with lower LDL-C concentrations and an increasing proportion of patients achieving the LDL-C target (Table 1). Taking multiple lipid-lowering agents was also associated with better control of systolic blood pressure (*p* = 0.002), possibly because the number of lipid-lowering agents serves as a surrogate marker for more intensive cardiovascular risk factor management in general, including the use of antihypertensive medication. Quality of life as measured by EuroQol (EQ-5D-5L) was not affected (health-related quality of life (EQ-VAS scale), *p* = 0.13; Table 1). Patients on combination LLT were more likely to have experienced elevations of creatine kinase or liver enzymes under statin therapy (Fig. Panel D). Furthermore, patients with a history of premature cardiovascular events or early revascularization were more likely to be treated with multiple lipid-lowering agents (Fig. Panel D) and reached their LDL-C goal more often (23.9 % vs. 11.7 %, *p* < 0.001).

**Figure:**
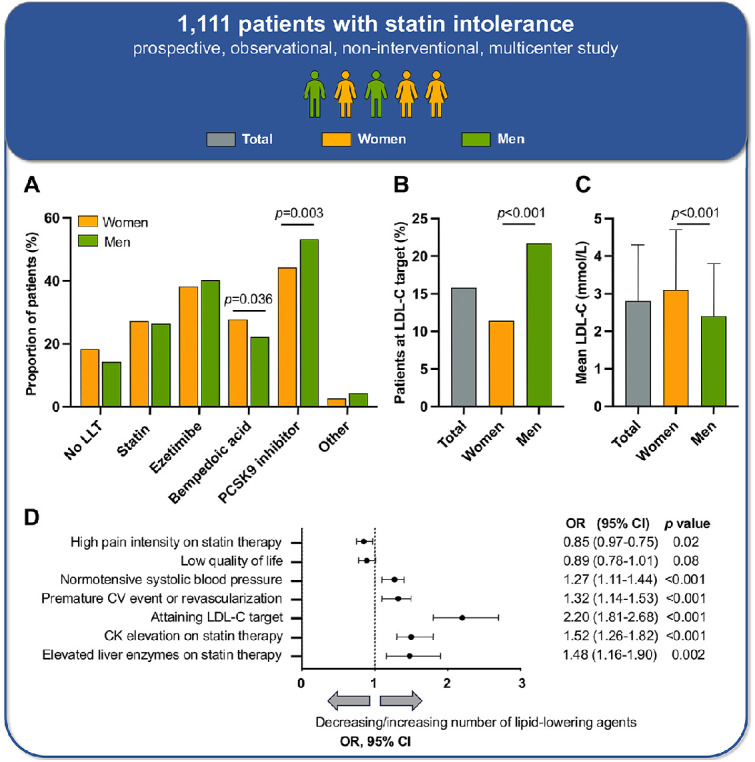
**Central illustration**. Panel A: Lipid-lowering agents at study inclusion. Distribution of the four most frequently taken lipid-lowering agents stratified by sex. “Other” encompasses a total of 5 patients on lipoprotein apheresis, 9 patients on fibrates, 7 patients on a bile acid sequestrants and 21 patients on another lipid-lowering therapy not specified. LLT: lipid-lowering therapy. Panel B and C: Sex differences in LDL-C serum concentrations and LDL-C target attainment LDL-C: low-density lipoprotein cholesterol. Panel D: Associations of the number of lipid-lowering agents with selected variables CI: confidence interval, CK: creatine kinase, CV: cardiovascular; LDL-C: low-density lipoprotein cholesterol, OR: odds ratio.

## 4 Discussion

This large cohort of patients with SI provides a contemporary characterization of the treatment strategies and LDL-C target attainment in this difficult-to-treat population. Most patients with SI received at least one LLT at inclusion (83.1 %), and 47.0 % were on combination LLT. Women achieved the LDL-C target to a much lower extent than men. An increasing number of lipid-lowering agents was associated with better control of LDL-C, more often with elevations of creatine kinase and liver enzymes under statin therapy, but no impairment in quality of life.

Due to the lack of an unambiguous diagnostic marker for SI, different definitions of SI have been proposed [6,8,9]. It is reassuring that randomized studies, the current registry, and previous simulation studies in patients with SI identified similar patient populations with regard to baseline characteristics [10,11,18]. The finding of PCSK9i prescriptions in more than half of the cases is consistent with the results of the German registries PEARL and PERI-DYS, which characterized patients treated with PCSK9i and found that approximately 70 % had SI [19,20]. The use of bempedoic acid was relatively low, considering that a recent simulation study indicated that half of the patients with SI could achieve the LDL-C target with bempedoic acid and ezetimibe [18]. Access to novel lipid-lowering therapies, including PCSK9i and bempedoic acid, can vary significantly between countries due to differences in healthcare systems, payer policies, and regulatory approvals. These factors might affect the generalizability of our findings.

Despite current recommendations for early combination LLT [21] to achieve the LDL-C target, more than half of the patients received no or only one lipid-lowering drug at baseline. Patients with a history of premature cardiovascular events or revascularization were more likely to receive multiple lipid-lowering which translated into improved LDL-C target achievement. The diagnosis of SI requires extra time and effort of the treating physicians. The low proportion of patients, especially women, attaining the LDL-C target despite a mean of 6.8 physician visits in the past year related to hyperlipidaemia is concerning and underscores the strong and unmet medical need to develop better strategies for patients with SI. Individualized treatment strategies might be a possible approach to optimize patient trajectories of SI patients [10]. Several studies, including large registries and retrospective analyses, have highlighted the undertreatment and missed or delayed diagnosis of dyslipidemia in women compared to men, driven by a range of factors, including sex-specific biological differences and gender-related socio-cultural aspects [[22], [23], [24], [25], [26], [27]]. Women are more susceptible to SI than men, report higher intensity of statin-associated muscle symptoms, and have worse quality of life than men with SI [3,10]. The current analysis shows that women with SI have significantly lower rates of LDL-C target achievement. Our results suggest an underestimation of ASCVD risk, show a striking undertreatment, and strongly suggest that special attention is needed to establish effective LDL-lowering treatment in women with SI.

Continuing statin therapy after reported adverse reactions has been associated with reduced cardiovascular risk [28]. Against our expectation, the number of different statins taken in the past was associated with a higher likelihood of statin therapy at baseline. This encouraging finding suggests that despite a history of several attempts of establishing statin therapy, trying another statin, preferably at a lower dose, may be a worthwhile option. More than one quarter of patients took a statin at baseline, which was associated with a significant and clinically relevant lower LDL-C concentration and higher rate of LDL-C target attainment. Our findings therefore support the clinical strategy to re-expose patients to a statin therapy despite previous SI [29].

## 5 Conclusion

In conclusion, in patients with SI, the use of a greater number of lipid-lowering medications, including statins, was not associated with reduced quality of life but with lower LDL-C concentrations. LDL-C target attainment was low, especially among women. Our results encourage a clinical strategy of repeated attempts of establishing some form of statin therapy and combination LLT in patients with SI.

## Ethical review statement

Each local Ethics Committee approved the study, written informed consent was obtained from all participants.

## Funding

This study was funded by Leipzig University and research grants from Daiichi Sankyo, Novartis, and Amgen to Leipzig University. The funding sources had no part in study design, data collection, data analysis, data interpretation, or writing of the report.

## CRediT authorship contribution statement

**Paulina E. Stürzebecher:** Writing – original draft, Visualization, Software, Methodology, Investigation, Formal analysis, Conceptualization. **Julius L. Katzmann:** Writing – original draft, Visualization, Methodology, Formal analysis. **Ionna Gouni-Berthold:** Writing – review & editing, Validation, Resources, Project administration, Investigation. **Christina Mateev:** Writing – review & editing, Validation, Investigation, Data curation. **Ole Frenzel:** Writing – review & editing, Validation, Investigation, Data curation. **Ulrike Schatz:** Writing – review & editing, Validation, Resources, Investigation, Data curation, Conceptualization. **Andrea Baessler:** Writing – review & editing, Validation, Supervision, Resources, Investigation. **Wolfgang Koenig:** Writing – review & editing, Validation, Supervision, Resources, Investigation, Data curation. **Stephan H. Schirmer:** Writing – review & editing, Validation, Supervision, Resources, Investigation, Data curation. **Irina Müller-Kozarez:** Writing – review & editing, Validation, Investigation, Data curation. **Oliver Weingärtner:** Writing – review & editing, Validation, Supervision, Resources, Investigation, Data curation, Conceptualization. **Ursula Kassner:** Writing – review & editing, Validation, Supervision, Resources, Investigation, Data curation, Conceptualization. **Ulrich Laufs:** Writing – original draft, Visualization, Validation, Supervision, Resources, Project administration, Methodology, Investigation, Funding acquisition, Data curation, Conceptualization.

## Declaration of competing interest

PES has received honoraria for lectures from Daiichi Sankyo, Amgen, Novartis, Synlab and travel grants from Daiichi Sankyo. IGB has received consulting honoraria from Amgen, Regeneron, Sanofi, Aegereon, Akcea Therapeutics, Novartis, Daiichi-Sankyo, Synlab, Ultragenyx and Amarin. JLK has received consulting fees and travel grants from Daiichi Sankyo. US has received honoraria from Amgen, Amarin, AstraZeneca, Boehringer Ingelheim, Daiichi Sankyo, Sanofi, Synlab, Novartis, NovoNordisk. AB reports advisory board fees from AMGEN, Sanofi, Daiichi Sankyo, Novartis, Amarin, Pfizer and honoraria/travel grants from Sanofi Aventis, AMGEN, Novartis, Daiichi Sankyo, Amarin, Pfizer, Bristol Myers Squibb. WK reports advisory board fees from AstraZeneca, Novartis, Amgen, Pfizer, The Medicines Company, DalCor, Kowa, Corvidia, OMEICOS, Daiichi-Sankyo, Novo Nordisk, New Amsterdam Pharma, TenSixteen Bio, Esperion, Genentech and LIB therapeutics; lecture fees from Bristol-Myers Squibb, Novartis, Amgen, Berlin-Chemie, Sanofi and AstraZeneca; grants and non-financial support from Abbott, Roche Diagnostics, Beckmann, and Singulex. SHS has received honoraria from Amgen, AstraZeneca, Bayer, Boehringer, Daiichi-Sankyo, MSD, Novartis, NovoNordisk, Pfizer, Sanofi, Synlab. IMK reports advisory board fees from Sobi and honoraria from Daiichi Sankyo. OW reports honoraria for lectures from AMGEN, Berlin-Chemie, Daiichi-Sankyo, Novo Nordisk, Novartis, Sanofi-Aventis, Fresenius, Hexal, Akcea Therapeutics and honoraria for advisory board activities from AMGEN, Sanofi-Aventis, Berlin-Chemie, Novartis, Hexal, Akcea Therapeutics, Daiichi-Sankyo, Pfizer and Sobi. UK has received honoraria from Daiichi Sankyo, Amgen, MSD, Synlab. UL has received honoraria from Amgen, AstraZeneca, Bayer, Boehringer, Daiichi-Sankyo, Lilly, MSD, Novartis, NovoNordisk, Pfizer, Sanofi, Synlab. CM, OF, have nothing to declare.
